# Analysis of network disruption evolution of Chinese fresh cold chain under COVID-19

**DOI:** 10.1371/journal.pone.0278697

**Published:** 2023-01-26

**Authors:** Huanwan Chen, Guopeng Chen, Qingnian Zhang, Xiuxia Zhang

**Affiliations:** 1 School of Transportation and Logistics Engineering, Wuhan University of Technology, Wuhan City, Hubei Province,China; 2 Planning and Operation Department, Hanjiang Water Conservancy & Hydropower Group Co., Ltd,Wuhan City, Hubei Province,China; 3 School of Modern Posts, Nanjing University of Posts and Telecommunications,Nanjing City, Jiangsu Province,China; Galgotias University, INDIA

## Abstract

The spread of the global COVID-19 epidemic, home quarantine, and blockade of infected areas are essential measures to prevent the spread of the epidemic, but efforts to prevent and control the outbreak lead to the disruption of fresh and cold chain agricultural products in the region. Based on the multi-layer management model of non-scale agricultural households in China, we applied the complex network theory to construct an evolutionary model of the Chinese fresh cold chain network with adaptation degree priority connection, dual local world considering transport distance connection relationship, and superiority and inferiority mechanism. Based on this model, we studied the evolution of fresh cold chain disruption, and puts forward the optimal design of fresh cold chain network disruption and reconnection from the perspective of practicality and economy.

## Introduction

The spread of the global new crown epidemic, home quarantine, and blockade of infected areas are essential measures to prevent the spread of the epidemic, but efforts to prevent and control the outbreak lead to disruption of fresh cold chain agricultural products in the region. There are many researches on supply chain network disruption, but the research on fresh cold chain network disruption is relatively few. In 2004, Thadakamalla et al. [1] proposed a structural model of supply chain network, while Z. Gang et al. [2] studied the characteristics of the agri-food supply chain network in inland China using complex network theory, and showed contradictory structural characteristics from other social networks. On the other hand, Yang Li et al. [3] constructed a distance-constrained agri-food supply chain evolving network model based on BA model [4]. Ghadge et al. [5] proposed a growth model for networks based purely on fitness; which had more flexibility and was able to define agri-food network topologies with fixed global properties. Nguyen and Tran [6]determined the direction of the values of the node fitness and constructed a growth model based on the connection probability which was directly proportional to the product of the degree of nodes and fitness. Smolyarenko [7] argued that the node connection probability should not entirely depend on the node degree, and proposed the node “fitness” concept, which merges the selection preferences of a given node. Bell et al. [8] showed that evolving networks with adaptability-based connections could be used in hierarchical heterogeneous supply chain networks. In addition, Perera et al. [9] proposed that supply chain network modeling should use empirical studies to identify key features in the topology of growth networks.

Many literatures have studied the evolution of supply chain network disruption and the relationship between network structure characteristics from the perspective of random attack and target attack, while there are few studies on the evolution of fresh cold chain network disruption. Nair [10] discussed the relationship between topology and network robustness of supply networks under random attacks and target attacks. From the theoretical analysis, it is concluded that the structural characteristics of the complex supply chain network are related to the network robustness. Further simulation and analysis of the random demand change and performance change of scale-free network and random network under random attack and target attack show that there is a significant correlation between the network characteristics and the robustness of supply network. Hiroyasu [11] studied the transmission of negative shocks in the supply chain network and found that: from a short-term perspective, the network structure seriously affects the transmission speed; From a long-term perspective, the network structure seriously affects the total loss. Enterprises with large connectivity conduct faster than those with small connectivity when the impact scale is the same. The greater the substitutability of supply chain network, the greater the negative impact transmission. The simulation results show that the direct damage caused by negative impact is different with different supply chain network structures. Yuhong Li [12] measures the network robustness after disruption by the ratio of the number of effective nodes, the proportion of the maximum connected graph, and the network efficiency. This paper analyzes how network structure and node risk capability affect different aspects of supply chain network elasticity. The results show that improving the node capacity is more effective than adjusting the network structure to improve the robustness of the supply chain network. Xiaoqiu Shi [13] proposed a cascaded fault model considering both functional and structural cascaded faults. The numerical simulation results show that different network types, interconnection modes and disruption types will seriously affect the robustness of the circular supply chain network; And the more uniform the distribution of network nodes in different network characteristics, the better the robustness of the circular supply chain network.

Inland fresh agricultural products of China have a large number of participants in each link, from harvesting (slaughtering or fishing) at the source to consumers via processing, storage, transportation, and distribution. According to the third national agricultural census, China has 314 million agricultural productions and operation personnel, 207.43 million agricultural operation households, 2.04 million agricultural operation units, and 1.79 million industrial and commercial registered cooperatives of farmers is such a huge scale of suppliers. In contrast, the spare parts suppliers of industrial products are relatively limited. In Chinese agricultural households, there were 3.98 million large-scale agricultural households, and more than 98%non-scale agricultural households. Z. Gang et al. [2] showed contradictory structural characteristics from other social networks. Yang Li et al. [3] thought that China’s agricultural product network can be divided into four layers, but they studied the robustness of node enterprises in the network from the perspective of competition. From the perspective of the circulation of agricultural products, we constructs a multi-level complex network of fresh cold chains with distinct levels. Combined with the operating characteristics of non-scale agricultural households, the network construction needs to consider the connection distance and level restrictions of node enterprises, and the adaptability of node selection. Studying the fresh cold chain network with the characteristics of non-scale agricultural households in China, research on the evolution of network disruption is helpful to analyze the healthy development of the fresh cold chain network in China.

## Methods

The data were obtained by setting the relevant parameters of the model to conduct numerical example analysis. The data was stored at (https://osf.io/su5kv/).

### Fresh cold chain network model construction

The following mechanisms need to be considered for the Chinese inland fresh cold chain network constructed by the article based on complex network theory.

1. Multi-layer structure. The fresh agricultural products supply chain of non-scale agricultural households in China is a typical push supply chain with many levels layers, scattered market players, loose relationship and low integration. Therefore, there is need for construction of a hierarchical supply chain network.
2. Local world. The technology of the Chinese inland fresh cold chain is inadequate, and the timeliness, loss rate, and transportation cost of fresh transportation restrict the scope of enterprises’ transactions, and the constructed network node connection has local limitations. The supply and demand relationship between entities is only related to the neighboring tiers and does not affect the entities at the same tier. [5]
3. Fitness. The preference of enterprises to choose transactions. The connection of nodes considers the preferences of fresh cold chain network entities based on their local transactions. Adaptability is defined as a comprehensive indicator of a node’s ability to attract nodes from the neighboring tiers. [6] The degree of adaptation is calculated as shown:
  $$\begin{array}{c} \eta_{i}=\alpha_{1}A_{i}+\alpha_{2}B_{i}+\ldots +\alpha_{m}M_{i} \end{array}$$ (1)
  In Eq (1), *A*_*i*_, *B*_*i*_, …, *Mi* denote the measure of node adaptation i, while *α*_1_, *α*_2_, …, *α*_*m*_ are the weights of selection preferences, *α*_1_+ *α*_2_+ …+ *α*_*m*_ = 1.
4. Growth and exit mechanism. The fresh cold chain system consists of an initial stage with a small number of nodes which form a simple structure, which then grows to new agricultural products nodes and edges, and then gradually evolve into a complex network. In the fresh agricultural products cold chain network, there is increased competition which often leads to elimination of some node enterprises from the network.

### Relevant description of the model

According to the relevant theory of graph theory, a network is a graph G = (V, E) consisting of an edge set *E* and a point set *V* where *E* = {(*v*_*i*_, *v*_*j*_)∣*v*_*i*_, *v*_*j*_∈*V*, *v*_*i*_≠*v*_*j*_}. We proposed in this article to construct a fresh cold chain network, where network nodes represent the physical enterprises, the edges represent the cooperative relationship between the physical enterprises, and the arrow direction is the transmission of the traded products from upstream to downstream. The cold chain network of fresh agricultural products, with the core enterprise as the node, is divided into four levels: farmer level, dealer level, wholesaler level and retailer level. Among them, the farmer level are non-scale agricultural households, the dealer level includes processors, purchasers, cooperatives, etc., the wholesaler level includes the origin wholesale market, the sales wholesale market, etc., and the retailer level includes retail stores, farmers’ markets, chain supermarkets, etc. These enterprises are controlled through logistics, information flow and capital flow. The description of the main symbols are shown in Table 1.

**Table 1:** Description of the main symbols.

Symbols	Description
*V*	The set of noeds that make up the network
*E*	The set of edges that make up the network
*T*	Hierarchy of networks T = 1,2,3,4
*V*_*T*_	The set of nodes at each level of the network *T*=1,2,3,4. For example: when *T*=1, then *V*_1_ is the set of nodes at first level (farmer level).
*v*_*i*_	A node of the network,*v*_*i*_ ∈ *V*
*e*_*ij*_	An edge of the network. Edge *e*_*i*_ *j* indicates that there is a edge from node *v*_*i*_ to node *v*_*j*_. If the node *v*_*i*_ and the node *v*_*j*_ have an interaction, the two nodes share an edge *e*_*ij*_, *v*_*i*_ and *v*_*j*_ are the two endpoints of *e*_*ij*_, which we mark as *e*_*ij*_ =1. If node *v*_*i*_ and node *v*_*j*_ do not have an interaction, the two nodes do not have a edge, which we mark as *e*_*ij*_ = 0.
*w*_*ij*_	The weight of the edge of the network indicates the transaction volume of the fresh cold chain brick-and-mortar companies.
*k*_*i*_	Degree of node *v*_*i*_
$k_{i}^{in}$	In-degree of node *v*_*i*_, the number of edges connecting the node *v*_*i*_ from other nodes.
$k_{i}^{out}$	Out-degree of node *v*_*i*_, the number of edges connecting other nodes from this node *v*_*i*_.
*s*_*i*_	Total strength of node *v*_*i*_
$s_{i}^{in}$	The weight of edges connecting the node *v*_*i*_ from other nodes, the node *v*_*i*_ into the strength.
$s_{i}^{out}$	The weight of edges connecting other nodes from this node *v*_*i*_, the node *v*_*i*_ out of strength.
*η*_*i*_	Adaptation of node *v*_*i*_
(*x*_*i*_, *y*_*i*_)	Location of node *v*_*i*_
*d*_*ij*_	Distance of node *v*_*i*_(*x*_*i*_, *y*_*i*_) and *v*_*j*_(*x*_*i*_, *y*_*i*_).
*D*_*T*_	Distance local limit between two levels, T = 1,2,3,4
*N*	Total number of nodes in the network
*N*_*n*_	Total number of nodes generated during the network construction
*V*_*b*_	The set of nodes to be deleted from the network
*E*_*b*_	The set of edges to be deleted from the network
*V*_*u*_	The set of failed nodes after network disruption
*E*_*u*_	The set of failed edges after network disruption
*V*_*f*_	The set of effective nodes after network disruption
*V*_*fT*_	The set of effective nodes at each level of the network after network disruption, T = 1, 2, 3, 4.
*E*_*f*_	The set of effective edges after network disruption
*E*_*fT*_	The set of effective edges at each level of the network after network disruption, T = 1, 2, 3, 4.
λ_*i*_	The load factor that can be added to the node
*Q*_*i*_	Maximum load capacity of node *v*_*i*_
*Q*	Total network strength

1. The relationship of node sets in the network. *V*_1_ is the first level farmer node set, *V*_2_ is the second level dealer node set, *V*_3_ is the third level wholesaler node set, and *V*_4_ is the fourth level retailer node. The relationship between the four hierarchical node sets *V*_1_, *V*_2_, *V*_3_, *V*_4_, and the network node set *V* is shown in Eq (2).
  $$\begin{array}{c} V=V_{1}\cup V_{2}\cup V_{3}\cup V_{4} \end{array}$$ (2)
2. The relationship of network node *v*_*i*_ degree (*k*_*i*_). The in-degree of node *v*_*i*_ ($k_{i}^{in}$)can be expressed by Eq (3), *e*_*ji*_ =1 means there is a edge from node *v*_*j*_ to node *v*_*i*_. The out-degree of node *v*_*i*_ ($k_{i}^{out}$)can be expressed by Eq (4), *e*_*ij*_ =1 means there is a edge from node *v*_*i*_ to node *v*_*j*_. So the degree of node *v*_*i*_ can be expressed by Eq (5).
  $$\begin{array}{c} k_{i}^{in}=\sum {}_{e_{ji}=1}^{j}e_{ji} \end{array}$$ (3)
  $$\begin{array}{c} k_{i}^{out}=\sum {}_{e_{ij}=1}^{j}e_{ij} \end{array}$$ (4)
  $$\begin{array}{c} k_{i}=k_{i}^{in}+k_{i}^{\text{out}\;} \end{array}$$ (5)
3. Network node strength (*s*_*i*_) relationship:
  $$\begin{array}{c} s_{i}^{in}=\sum_{\begin{array}{c} k\in V\\ e_{ji}=1 \end{array}}^{k_{i}^{in}}w_{ji} \end{array}$$ (6)
  $$\begin{array}{c} s_{i}^{\text{out}\;\;}=\sum_{\begin{array}{c} j\in V\\ e_{ij}=1 \end{array}}^{k_{i}^{\text{out}}}w_{ij} \end{array}$$ (7)
  $$\begin{array}{c} s_{i}=s_{i}^{in}+s_{i}^{\text{out}\;} \end{array}$$ (8)
  $$\begin{array}{c} s_{i}^{in}=s_{i}^{\text{out}\;},\;(v_{i}\in V_{2}\;\text{or}\;v_{i}\in V_{3}) \end{array}$$ (9)
  Eq (9) indicates that the constructed network must consider the transaction volume balance, i.e., if node *v*_*i*_ is an intermediate node in the fresh cold chain network, the incoming strength of the node is equal to the outgoing strength of the node.

### Rules of the model

1. Network level setting After examining the fresh cold chain in inland China, we constructed a four-tier fresh cold chain network in this article.
2. Selection of fitness Fitness is a comprehensive indicator to reflect the selection preferences of nodes in the network. Different entities consider different preferences for connection. [14]. Whereas it is impractical to calculate the fitness of each node in the modeling, the fitness *η*_*i*_ is selected from relevant studies to obey a normal distribution with parameter $F\left(\eta_{i}\right)\propto N\left(\mu_{i},\sigma_{i}^{2}\right)$. [6–8]
3. Local-world network In real life, the class of network nodes does not attain a global priority connection. Constrained by the geographical differences, timeliness of fresh food transport, loss rate, and transportation cost among the node enterprises, the fresh cold chain network nodes have local restrictions when selecting connections. There is also existence of preferential connectivity in the local world (LW) [8]. Newly added nodes select *m*_*x*_ nodes to be connected according to the priority connection probability equation, LW is also known as the geographically restricted local world.(*m*_*x*_ represents the number of nodes selected for connection by new nodes at each level.)
  $$\begin{array}{c} \prod_{\text{local}\;}\left(j\to i\right)=\frac{\eta_{i}s_{i}}{\sum_{i\in LW}\eta_{i}s_{i}} \end{array}$$ (10)
  In the Eq (10), *η*_*i*_ denotes the fitness of node *v*_*i*_ while *s*_*i*_ shows the strength of node *v*_*i*_. *LW* is also the local domain in which a new node *v*_*j*_ can be selected. The probability that a particular node *v*_*i*_ is connected to a new node *v*_*j*_ depends on the ratio of the product of the strength and fitness of that node to the product of the degrees and fitness of all nodes in local domain.
4. Node growth
  The nodes in the fresh cold chain network emanate from different geographical locations, and the constraints of geographical location limit the node connections in the network. Assuming that the upstream and downstream tier nodes are evenly affected by geographic distance, the geographic distance between nodes *v*_*i*_(*x*_*i*_, *y*_*i*_) and nodes *v*_*j*_(*x*_*j*_, *y*_*j*_) of two adjacent tiers is expressed by *d*_*i*_ *j*. According to the differences in the service capacity of nodes in different tiers, the distance between the tiers is different due to the local geographical distance (*D*_*T*_), which restricts the distance between different tiers.

### Construction of the model

Each node generated in the model is randomly assigned the following four pieces of information: location (*x*_*i*_, *y*_*i*_), level to which the node belongs (*T*), adaptation degree (*η*_*i*_), and node strength (*s*_*i*_). Taking the four layer network as an example, the four hierarchical levels are: farmer (*T*_1_) → dealer (*T*_2_) → wholesaler (*T*_3_) → retailer (*T*_4_).

**Fig 1:**
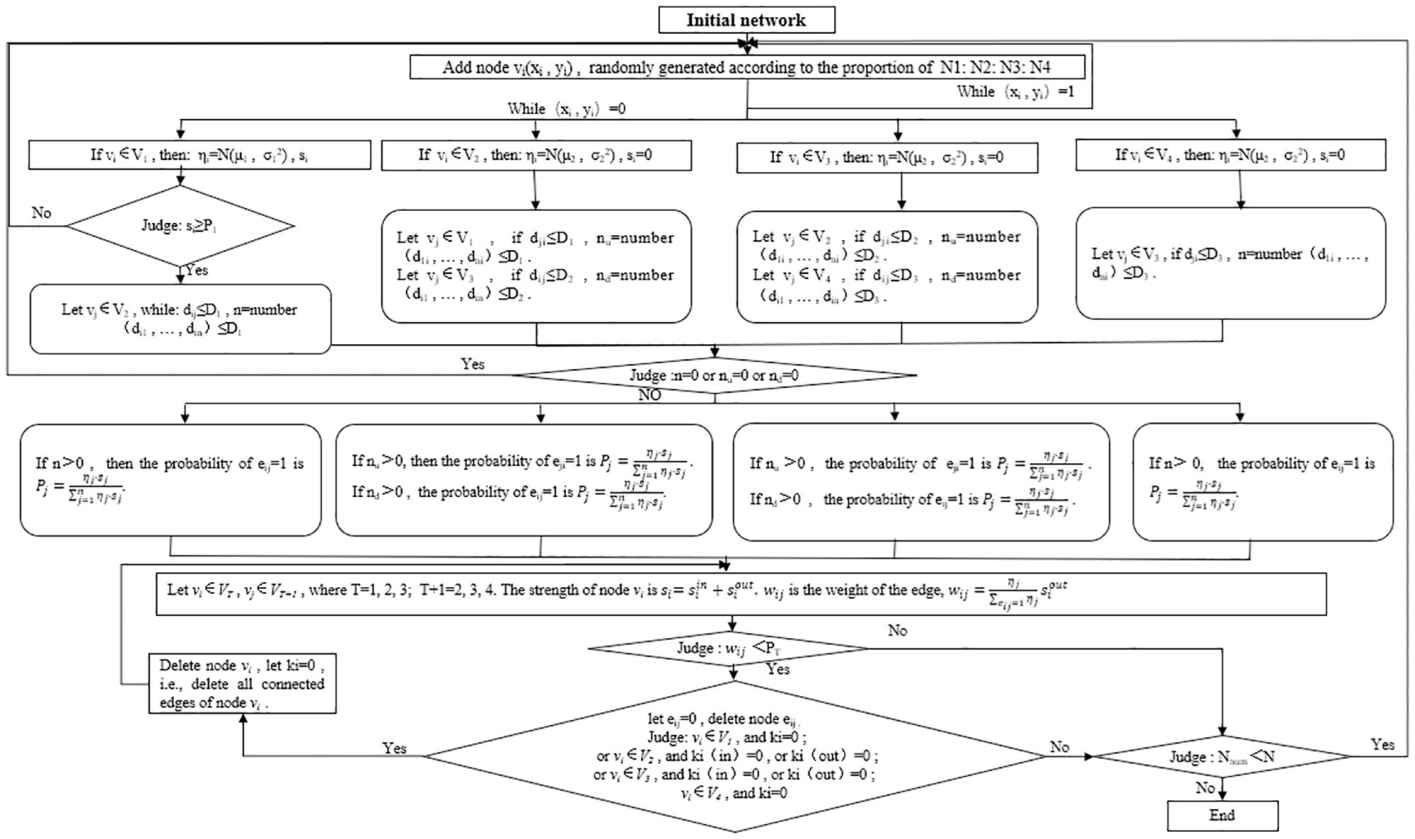
Model construction flow chart.

1. Initial network. When *t* = 0, the network starts with a four-level simple network containing *m*_0_ nodes and *e*_0_ connected edges.
2. The classes of nodes (i.e., the hierarchy in which the nodes are located) are randomly generated in the ratio of *N*_1_: *N*_2_: *N*_3_: *N*_4_.
  Initial network. When *t* = 0, it starts with a simple four-level network containing *m*_0_ nodes and *e*_0_ edges. The *e*_0_ directed edges in the network connect upstream and downstream of the adjacent levels, where the edges are connected in accordance with the distance restrictions: farmer (*T*_1_) is connected with dealer (*T*_2_) at a distance *d*_*ij*_ ≤ *D*_1_, dealer (*T*_2_) is connected with wholesaler (*T*_3_) at a distance *d*_*ij*_ ≤ *D*_2_, and wholesaler (*T*_3_) is connected with retailer (*T*_4_) at a distance *d*_*ij*_ ≤ *D*_3_. Each farmer (*T*_1_) is assigned an initial point according to node *v*_*j*_ intensity *s*_*j*_, and the connected dealer (*T*_2_) node *v*_*i*_ is assigned edge weights according to the fitness.
  $$\begin{array}{c} w_{ji}=\frac{\eta_{i}}{\sum_{eji=1}^{i}\eta_{i}}s_{j}^{\text{out}\;} \end{array}$$ (11)
  In Eq (11), keeping the sum of out-degree edge rights of dealer (*T*_2_) equal to the sum of in-degree edge rights, we allocated edge rights downstream of the supply chain to the retailer.
3. Growth of the network
  The classes of nodes (i.e., the hierarchy in which the nodes are located) are randomly generated in the ratio *N*_1_: *N*_2_: *N*_3_: *N*_4_. Denote the total number of nodes generated in the network construction process by *N*_*num*_, and N denotes the total number of nodes that reach the network size (Fig 1).

## Network disruption evolution model of fresh cold chain

Due to unexpected events, the connection between nodes in the fresh cold chain network is disrupted or the node disappears. According to the original connection relationship of the network, the network redistributes the node strength, which may occur again: the node lacks downstream connection and cannot transport fresh agricultural products to the network terminal, and the node lacks upstream and no fresh agricultural products can be traded. In order to fully understand the evolution of node disrupts in the network, it is necessary to build an disruption evolution model based on the fresh cold chain network model constructed in the previous section.

In reality, the capacity of each enterprise is limited. When the supply received by the enterprise exceeds the upper limit of the enterprise, it may lead to the overstock of the enterprise’s inventory and increase the risk of inventory realization, thus causing financial risks and causing operational failure. Therefore, the node load capacity of the model is limited. After the network node is disrupted, the network redistributes the node strength, and the nodes exceeding the load capacity will fail. The ability to maintain its function after network disruption is measured by relevant indicators to express the result of evolution after network disruption.

### Description of the model

1. Node load capacity
  Assuming that all nodes of the network have the same load capacity, the initial load of the nodes of the fresh cold chain network interruption evolution model is the node strength of the fresh cold chain network model. The load upper limit of the node is related to the original strength of the node, as shown Eq (12).
  $$\begin{array}{c} Q_{i}=\left(1+\lambda \right)s_{i} \end{array}$$ (12)
  In Eq (12): λ is the load factor, λ=0.1 0.2 0.3…1.
2. Generation of interrupt nodes
  The node interrupt can be divided into two cases: random interrupt node and target interrupt node. Randomly occurring node outages: Randomly delete a specified number of nodes from the network. Targeted node outage: network nodes are deleted as required. The order of target deletion: the ratio of node degrees to the total number of degrees of nodes in the tier to which they belong, and this ratio carries out the descending order of nodes in the whole network.
3. Evolution process of node interruption
  Take the constructed fresh cold chain network as the initial network, if the node is interrupted, the interrupted nodes and edges in the network are deleted. Because of the path dependence, according to the original network relationship and weight distribution, the network evolution process may appear: failure nodes with node overload, failure nodes that do not reach the minimum edge connection threshold, failure nodes that lack upstream and downstream connections. After the network deletes such nodes and edges, the network finally presents nodes and edges that can conduct effective fresh agricultural products transmission.

### Construction of interrupt evolution model

The load transmission process of network disruption is as follows.

➀ Constructing a fresh cold chain network model. The total number of nodes is *N*, the total number of edges is *M*, the average path of the network is *L*, the total number of supply chains is *R*, and the total volume of transactions circulating in the network is *Q*. According to Eq (13), the upper limit *Q*_*i*_ of a load of each node is set.

➁ Deleting the specified nodes *N*_*b*_ (edges *M*_*b*_) by random (target) node disruption. A random (target) node break with *v*_*i*_ ∈ *V*_*b*_ then *E*_*b*_ = {*e*_*ij*_∈*E* and *v*_*i*_∈*V*_*b*_}:

$$\begin{array}{c} N_{b}=\sum {}_{v_{i}\in V_{b}}1 \end{array}$$ (13)

$$\begin{array}{c} M_{b}=\sum {}_{v_{i}\in V_{b}}k_{i} \end{array}$$ (14)

➂ Determining the failed nodes and edges generated by the network and removing the failed nodes and edges. Judgment and deletion are performed from upstream to downstream of the network.

(A)First level generation: failed nodes

If *v*_*j*_ ∈ *V*_*l*_ and *v*_*j*_ ∉ *V*_*b*_, *v*_*j*_ ∉ *V*_*u*_, there exists *v*_*i*_ ∈ *V*_*b*_, *v*_*b*_ ∈ *V*_*u*_, and there are:

$$\begin{array}{c} k_{j}=\sum {}_{e_{ji}\in E_{b}}1+\sum {}_{e_{j\hbar }\in E_{u}}1 \end{array}$$ (15)

Then node *v*_*j*_ changes to a failed node, i.e., *v*_*j*_*V*_*u*_.

(B) Second and third level generation: failed nodes and failed edges

(a) If *v*_*j*_ ∈ *V*_2_(or *v*_*j*_∈*V*_3_) and *v*_*j*_ ∉ *V*_*b*_, *v*_*j*_ ∉ *V*_*u*_, there exists *v*_*i*_ ∈ *V*_*b*_, *v*_*h*_ ∈ *V*_*u*_, and there are:

$$\begin{array}{c} k_{j}^{in}=\sum {}_{e_{ij}\in E_{b}}1+\sum {}_{e_{hj}\in E_{u}}1\;\;\text{and}\;\;k_{j}^{\text{out}\;}>\sum {}_{e_{ji}\in E_{b}}1+\sum {}_{e_{j\hbar }\in E_{u}}1 \end{array}$$ (16)

Then node *v*_*j*_ changes to a failed node, i.e., *v*_*j*_ ∈ *Vu*, and adds failed edges *e*_*jl*_ ∈ *E*_*u*_(where *e*_*jl*_ ∈ *E* and nodes *v*_*l*_ ∉ *V*_*b*_, *v*_*l*_ ∉ *Vu*).

(b) If *v*_*j*_ ∈ *V*_2_(or *v*_*j*_∈*V*_3_) and *v*_*j*_ ∉ *V*_*b*_, *v*_*j*_ ∉ *V*_*u*_, there exists *v*_*i*_ ∈ *V*_*b*_, *v*_*h*_ ∈ *V*_*u*_, and there are:

$$\begin{array}{c} k_{j}^{in}>\sum {}_{e_{ij}\in E_{b}}1+\sum {}_{e_{hj}\in E_{u}}1\;\;\text{and}\;\;k_{j}^{\text{out}\;}=\sum {}_{e_{ji}\in E_{b}}1+\sum {}_{e_{j\hbar }\in E_{u}}1 \end{array}$$ (17)

Then node *v*_*j*_ changes to a failed node, i.e., *v*_*j*_ ∈ *Vu*, and adds failed edges *e*_*jl*_ ∈ *E*_*u*_(where *e*_*jl*_ ∈ *E* and nodes *v*_*l*_ ∉ *V*_*b*_, *v*_*l*_ ∉ *Vu*).

(c) If *v*_*j*_ ∈ *V*_2_(or *v*_*j*_∈*V*_3_) and *v*_*j*_ ∉ *V*_*b*_, *v*_*j*_ ∉ *V*_*u*_, there exists *v*_*i*_ ∈ *V*_*b*_, *v*_*h*_ ∈ *V*_*u*_, and there are:

$$\begin{array}{c} k_{j}^{in}=\sum {}_{e_{ij}\in E_{b}}1+\sum {}_{e_{hj}\in E_{u}}1\;\;\text{and}\;\;k_{j}^{\text{out}\;}=\sum {}_{e_{ji}\in E_{b}}1+\sum {}_{e_{j\hbar }\in E_{u}}1 \end{array}$$ (18)

Then node *v*_*j*_ changes to a failed node, i.e., *v*_*j*_ ∈ *Vu*, and adds failed edges *e*_*jl*_ ∈ *E*_*u*_(where *e*_*jl*_ ∈ *E* and nodes *v*_*l*_ ∉ *V*_*b*_, *v*_*l*_ ∉ *Vu*).

(C) Fourth level generation: failed nodes and failed edges

If *v*_*j*_ ∈ *V*_4_ and *v*_*j*_ ∉ *V*_*b*_ *v*_*j*_ ∉ *Vu*, there exists *v*_*i*_ ∉ *V*_*b*_, *v*_*h*_ ∈ *V*_*u*_, and there are:

$$\begin{array}{c} k_{j}=\sum {}_{e_{ij}\in E_{b}}1+\sum {}_{e_{hj}\in E_{u}}1 \end{array}$$ (19)

Then node *v*_*j*_ changes to a failed node, i.e., *v*_*j*_ ∈ *V*_*u*_, and adds failed edges *e*_*jl*_ ∈ *E*_*u*_(where *e*_*jl*_ ∈ *E* and nodes *v*_*l*_ ∉ *V*_*b*_, *v*_*l*_ ∉ *V*_*u*_).

Repeat (A) and (C) to remove the failed nodes and edges generated in the network until the network does not generate failed nodes and edges.

➃ Network edge power evolution.

Denote the set of effective nodes after network disruption (*V*_*f*_) and the set of effective edges after network disruption (*E*_*f*_). Then we have:

$$\begin{array}{c} V=V_{f}\cup V_{b}\cup V_{u}\;\text{and}\;V_{f}\cap V_{b}=0\;,\;V_{f}\cap V_{u}=0\;,\;V_{u}\cap V_{b}=0 \end{array}$$ (20)

$$\begin{array}{c} E=E_{f}\cup E_{b}\cup E_{u}\;\text{and}\;E_{f}\cap E_{b}=0\;,\;E_{f}\cap E_{u}=0\;,\;E_{u}\cap E_{b}=0 \end{array}$$ (21)

Take *V*_*f*1_, *V*_*f*2_, *V*_*f*3_, and *V*_*f*4_ to denote the effective node sets at the first, second, third, and fourth levels of the network, respectively, and *E*_*f*1_, *E*_*f*2_, and *E*_*f*3_ to denote the effective set of effective edges between nodes at the four levels of the network from upstream to downstream, in that order. Then, the connected edge weights of the network are assigned as Eq (10) at this time, but the nodes and edges need to be satisfied: *v*_*i*_ ∈ *V*_*f*_, *v*_*j*_ ∈ *V*_*f*_, *e*_*ij*_ ∈ *E*_*f*_.

➄ Determining the weights of the valid valid edges *e*_*ij*_ ∈ *E*_*f*1_.

(A) If there exists *ω*_*ij*_ < *P*_1_, change the effectively edge *e*_*ij*_ to an invalid edge, i.e., change *e*_*ij*_ ∈ *E*_*f*1_ to *e*_*ij*_ ∈ *E*_*u*_,and remove the edge *e*_*ij*_from the network and repeat step ➂ to ➃ judge and remove the edge *e*_*ij*_ in the network that has been changed from *e*_*ij*_ ∈ *E*_*f*1_ to *e*_*ij*_ ∈ *E*_*u*_ because of *ω*_*ij*_ < *P*_1_, until the weights of *e*_*ij*_ ∈ *E*_*f*1_ are all *ω*_*ij*_ ⩾ *P*_1_, then enter (6)

(B) If any *e*_*ij*_ ∈ *E*_*f*1_ exists: *ω*_*ij*_ ⩾ *P*1, then enter ➅.

➅ Judge the incoming strength $s_{i}^{in}$ of the valid node *v*_*i*_ ∈ *V*_*f*2_ according to Eq (22).

$$\begin{array}{c} s_{i}^{in}=\sum {}_{e_{ji}\in E_{f}}^{v_{j}\in V_{f1}}w_{ji} \end{array}$$ (22)

(A) If there exists $s_{i}^{in}$ > *Q*_*i*_/2, then node *v*_*i*_ becomes an invalid node due to overload, i.e., let *v*_*i*_ ∈ *V*_*f*2_ change to *vi* ∈ *Vu*, and its edge *e*_*ij*_ will also change to the invalid edge, and delete node *v*_*i*_ and its edge *e*_*ij*_ from the network. Repeat steps ➂ to ➄ judge and delete vi and its edge *e*_*ij*_ in the network, which has become an invalid node due to $s_{i}^{in}$ > *Q*_*i*_/2, until *v*_*i*_ ∈ *V*_*f*2_ with strength $s_{i}^{in}⩽Q_{i}/2$, then enter ➆

(B) If any *v*_*i*_*V*_*f*2_ exists: $s_{i}^{in}⩽Q_{i}/2$ then go to ➆.

➆ Judge the weights of the effectively edges *e*_*ij*_ ∈ *E*_*f*2_.

(A) If there exists *w*_*ij*_ < *P*_2_, change the effectively edge *e*_*i*_ *j* to the invalid edge, i.e., change *e*_*i*_ *j* ∈ *E*_*f*2_ to *e*_*ij*_ ∈ *E*_*u*_, and remove the edge *e*_*i*_ *j* from the network. Repeat steps ➂ to ➅, judge and remove the edge *e*_*ij*_ in the network that has been changed from *e*_*ij*_ ∈ *E*_*f*1_ to *e*_*ij*_ ∈ *E*_*u*_ because of *w*_*ij*_ < *P*_2_, until the weights of *e*_*ij*_ ∈ *E*_*f*2_ and are all *w*_*ij*_ ⩾ *P*_2_, then proceed to ➇.

(B) If any *e*_*ij*_ ∈ *E*_*f*2_ is present:*ω*_*ij*_ ⩾ *P*_2_, then enter ➇.

➇ Judge the incoming strength $s_{i}^{in}$ of the valid node *v*_*i*_ ∈ *V*_*f*3_ according to Eq (23).

$$\begin{array}{c} s_{i}^{in}=\sum {}_{e_{ji}\in E_{f}}^{v_{j}\in V_{f2}}w_{ji} \end{array}$$ (23)

(A) If there exists $s_{i}^{in}>Q_{i}/2$, then node *v*_*i*_ becomes an invalid node due to overload, i.e., let *v*_*i*_ ∈ *V*_*f*3_ change to *v*_*i*_ ∈ *V*_*u*_, and its edge *e*_*ij*_ will also change to the invalid edge, and delete node *v*_*i*_ and its edge *e*_*ij*_ from the network. Repeat steps ➂ to ➄, judge and delete *v*_*i*_ and its edge *e*_*ij*_ in the network, which has become an invalid node due to $s_{i}^{in}>Q_{i}/2$, until *v*_*i*_ ∈ *V*_*f*_3 with strength $s_{i}^{in}⩽Q_{i}/2$, then proceed to ➈.

(B) If any *v*_*i*_ ∈ *V*_*f*3_ exists: $s_{i}^{in}⩽Q_{i}$/2, then proceed to ➈.

➈ Judge the weights of the effectively edges *e*_*ij*_ ∈ *E*_*f*3_ (A) If there exists *w*_*ij*_ < *P*_3_, change the effectively edge *e*_*ij*_ to an invalid edge, i.e., change *e*_*ij*_ ∈ *E*_*f*3_ *toe*_*ij*_ ∈ *E*_*u*_, and remove the edge *e*_*ij*_ from the network. Repeat ➂ to ➇, judge and remove the edge *e*_*ij*_ in the network that has been changed from *e*_*ij*_ ∈ *E*_*f*1_ to *e*_*ij*_ ∈ *E*_*u*_ due to *w*_*ij*_ < *P*_3_, until the weights of *e*_*ij*_ ∈ *E*_*f*2_ are all *w*_*ij*_ ≥ *P*_3_, then proceed to ➉.

(B) If any *e*_*ij*_ ∈ *E*_*f*3_ *exists*: *ω*_*ij*_ ≥ *P*_3_, then enter ➉.

➉ Judge the incoming strength $s_{i}^{in}$ of the valid node *v*_*i*_ ∈ *V*_*f*4_ according to Eq (24).

$$\begin{array}{c} s_{i}^{in}=\sum {}_{e_{ji}\in E_{f}}^{v_{j}\in V_{f3}}w_{ji} \end{array}$$ (24)

(A) If there exists $s_{i}^{in}>Q_{i}/2$, node *v*_*i*_ becomes an invalid node due to overload, i.e., let *v*_*i*_ ∈ *V*_*f*4_ change to *v*_*i*_ ∈ *V*_*u*_ and its edge *e*_*ij*_ change to the invalid edge, and delete node *v*_*i*_ and its edge *e*_*ij*_ from the network. Repeat ➂ to ➈ judge and delete vi and its edge *e*_*ij*_ in the network that has become an invalid node due to $s_{i}^{in}⩾Q_{i}/2$, until *v*_*i*_ ∈ *V*_*f*4_ with strength $s_{i}^{in}⩽Q_{i}/2$, then enter ⑪.

(B) If any *v*_*i*_ ∈ *V*_*f*4_ exists: $s_{i}^{in}⩽Q_{i}/2$, then enter ⑪

⑪ The counts the total number of effective nodes *N*_*f*_, the average path *L*_*f*_, the total number of supply chains *N*_*Rf*_, and the total number of transactions *Q*_*f*_ in circulation in the network at this time.

#### Networkability metrics

How to measure the performance of a network after random and targeted disruptions? The literature [15] measured the network’s resilience after disruption in terms of accessibility, robustness, flexibility, and responsiveness. Wang [16] used network efficiency to measure the robustness of network cascading failures. Zhang [17] used fraction of surviving nodes to measure the robustness of network cascading failures. Hosseinalipour [18] used number of surviving nodes to measure the robustness of network cascading failures. Yang [19] used fraction of failed node to measure the robustness of network cascading failures. We explored the changes in the performance of the fresh cold chain network after disruption compared with the original network in four aspects: the proposed effective node ratio P(N), the average path length ratio P(L), the supply chain number ratio P(R), and the end retailer weight ratio P(Q). (1) The ratio of the number of effective nodes in the network

The relationship of the network node is set after interruption. Then the relationship between the four hierarchical node sets *V*_*f*1_, *V*_*f*2_, *V*_*f*3_, *V*_*f*4_, and the network node set *V*_*f*_ is shown in Eq (25).

$$\begin{array}{c} V_{f}=V_{f1}\cup V_{f2}\cup V_{f3}\cup V_{f4} \end{array}$$ (25)

Effective nodes are the nodes in the network that do not exceed the node load and have upstream and downstream connectivity to conduct normal product transactions. The number of effective nodes covered by the network indicates that the larger the network coverage, the better the connectivity.

The ratio of the effective number of nodes in the network is calculated by Eq (26).

$$\begin{array}{c} P\left(N\right)=\frac{N_{f}}{N} \end{array}$$ (26)

Where *N* is the total number of nodes in the original network and *N*_*f*_ is the total number of effective nodes after the network disruption.

(2) Network average path change ratio

The average path L of the network is a measure of network efficiency. The article used the ratio of the change in the average path length L before and after the network disruption to measure the change in network efficiency. The average path length L of the network is defined as the average distance between any two nodes.

$$\begin{array}{c} L=\frac{2}{N(N+1)}\sum_{i\geq j}d_{ij} \end{array}$$ (27)

The network average path ratio is calculated by Eq (28).

$$\begin{array}{c} P\left(L\right)=\frac{L_{f}}{L} \end{array}$$ (28)

Where *L* is the average path in the original network and *L*_*f*_ is the effective average path after the network disruption.

(3) Network supply chain change ratio

The number of supply chains in the network can reflect the complexity of the network. The r denotes the network supply chain, and *N*_*R*_ denotes the total number of network supply chains, expressed as Eq (29).

$$\begin{array}{c} P\left(R\right)=\frac{N_{R_{f}}}{N_{R}} \end{array}$$ (29)

Where *N*_*R*_ is the total number of supply chains in the original network and *N*_*Rf*_ is the total number of supply chains after the network disruption.

(4) Network weight change ratio

With the original load unchanged, the change in weights (transaction volume) can visualize the circulation performance of the network due to the disruption of the network resulting in a decrease in the weights (transaction volume) received by the nodes in the fourth tier. The network constructed in this paper is an entitled network, so this paper utilized the probability of network weight change as one of the indicators to measure the network robustness.

$$\begin{array}{c} P\left(Q\right)=\frac{Q_{f}}{Q} \end{array}$$ (30)

Where *Q* is the total weights in the original network and *Q*_*f*_ is the total weights after the network disruption.

## Example analysis

Due to the limitations of the survey data, we try to use different network structures to compare and analyze the network disruption evolution of the fresh cold chain in order to increase the reliability of the network research.

### Constructing a fresh cold chain network model

1. Given the initial network. As the network evolves, the network scale grows, and the initial network has less and less influence on the evolutionary model. In this paper, an initial network with *m*_0_ and *e*_0_ is given. Given an initial network with *m*_0_ = 20, *e*_0_ = 26.
2. The node adaptation degree is set. The fitness of farmer *η*_1_: F(*η*_1_) ∝ N(5, 1) The fitness of dealer *η*_2_: F(*η*_2_) ∝ N(200, 50^2^) The fitness of wholesaler *η*_3_: F(*η*_3_) ∝ N(500, 100^2^) The fitness of retailer *η*_4_: F(*η*_4_) ∝ *N*(4, 1).
3. Setting of node positions: position coordinates (x, y coordinates), {*x*, *y* ∈ (0, 10000)}
4. Edge localization setting: *D*_1_ = 3000, *D*_2_ = 4000, *D*_3_ = 2000.
5. Four levels of node ratio settings. Set two different ratios for comparative analysis of network interruption: Practice: *N*_1_: *N*_2_: *N*_3_: *N*_4_ = 40: 4: 1: 5 and Contrast: *N*_1_: *N*_2_: *N*_3_: *N*_4_ = 9: 3: 2: 6.
6. Newly joined nodes with edge settings. $m_{1}=2,m_{2}^{u}=4,m_{2}^{d}=2,m_{3}^{d}=3,m_{3}^{d}=4,m_{4}=2$ and $m_{2}=m_{2}^{u}+m_{2}^{d},m_{3}=m_{3}^{u}+m_{3}^{D}$.
7. The threshold of the edge is set. *P*_1_ = 90, *P*_2_ = 100, *P*_3_ = 40.
8. Nodal strength of farmers is set. The node strength of the farmer is related to its adaptation degree, and *s*_*i*_ = 100*η*_*i*_ is set.

According to the set parameters, we got the network data of Practice S1 Table and Contrast S2 Table.

### An example of network disruption evolution in fresh cold chain

According to the data of S1 and S2 Tables, we conduct interrupt evolution analysis on (Practice) network and (Contrast) network.

(1) Random disruption analysis

To express the graph and table data easily, Del-N indicates the number of nodes attacked on the deletion network, such as Del-1 indicates the deletion of one attacked node; Del-N% indicates the proportion of nodes attacked on the deletion network, such as Del-10% indicates the deletion of 10% of nodes attacked on the network. The following charts with such expressions have a consistent meaning.

**Fig 2:**
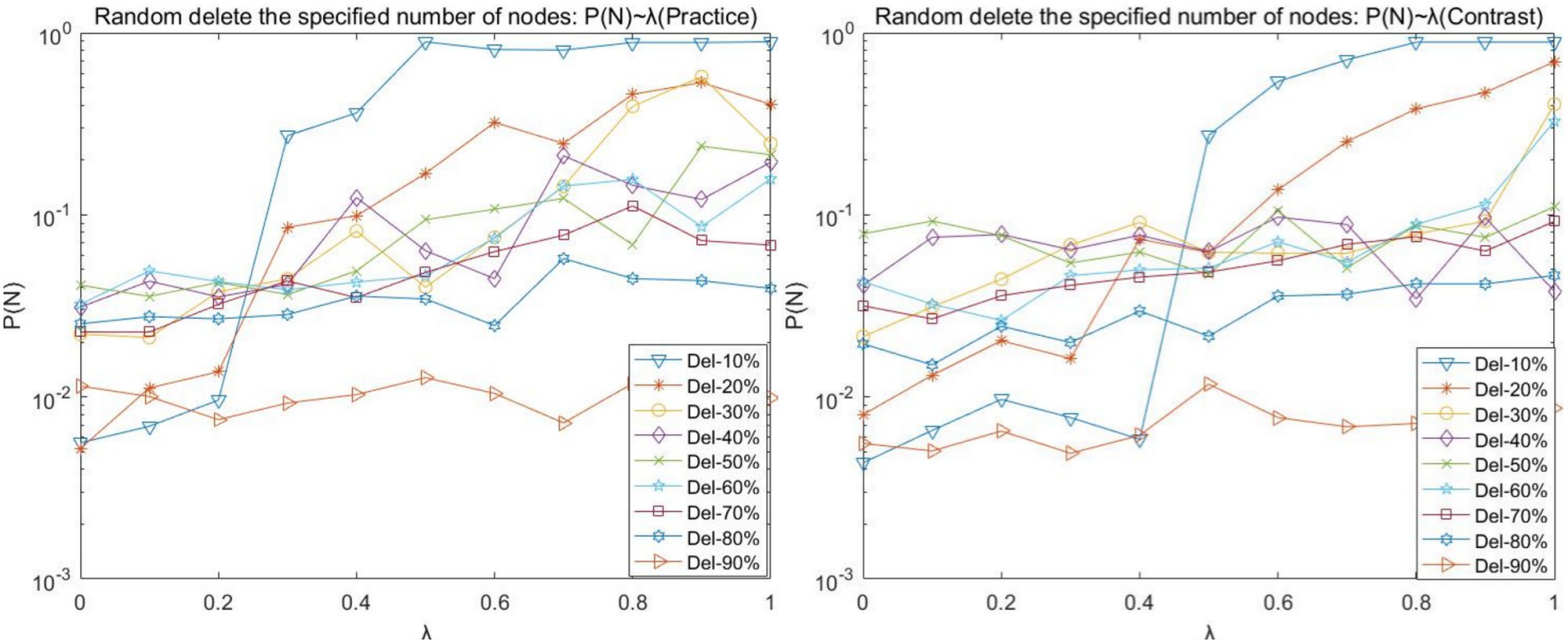
Random disruption- *P*(*N*) with λ variation diagram.

**Fig 3:**
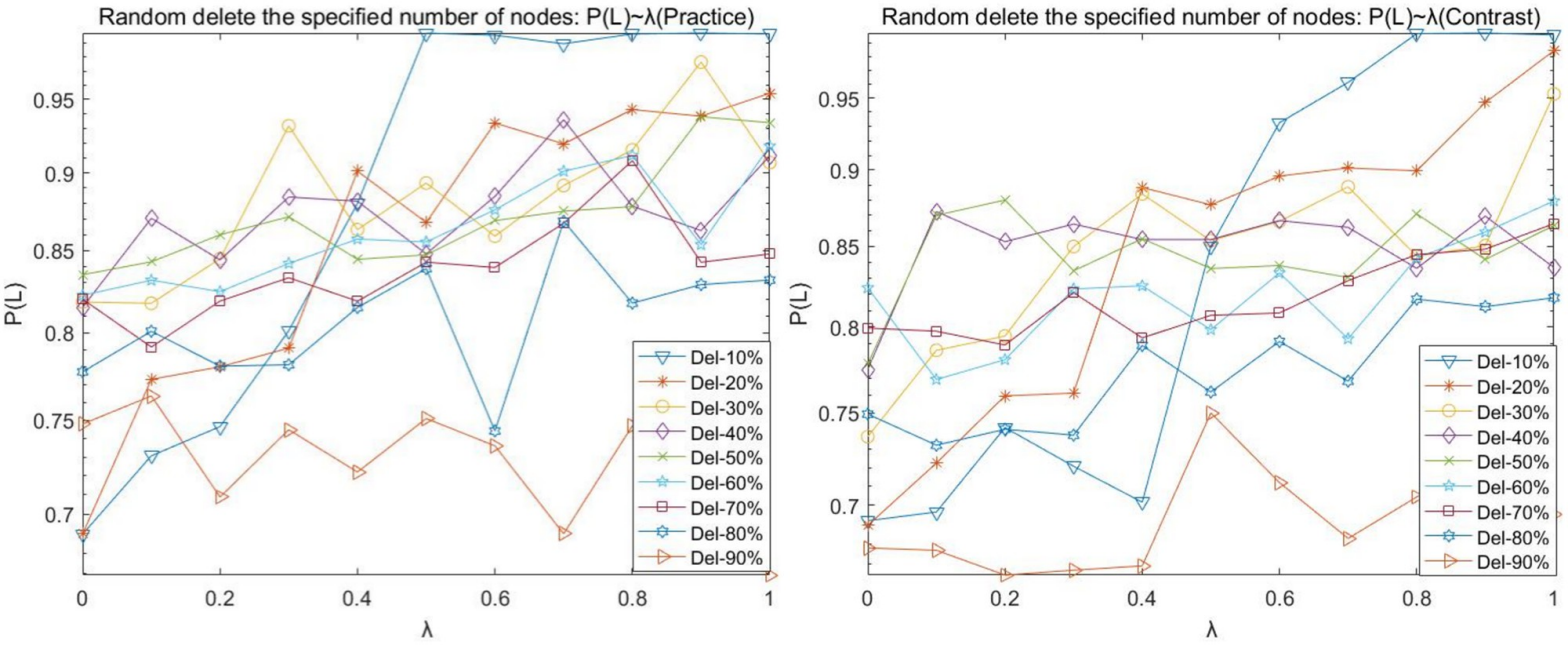
Random disruption- *P*(*L*) with λ variation diagram.

**Fig 4:**
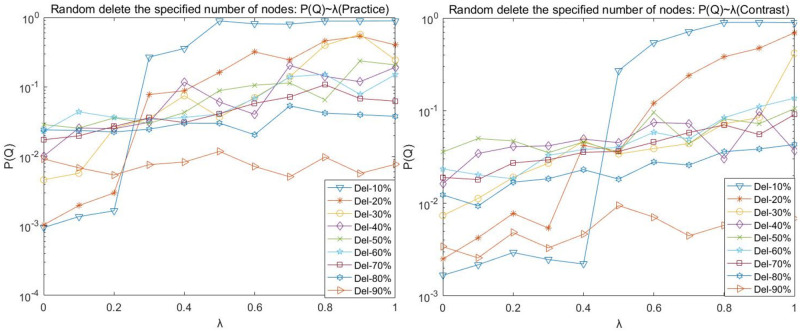
Random disruption- *P*(*Q*) with λ variation diagram.

**Fig 5:**
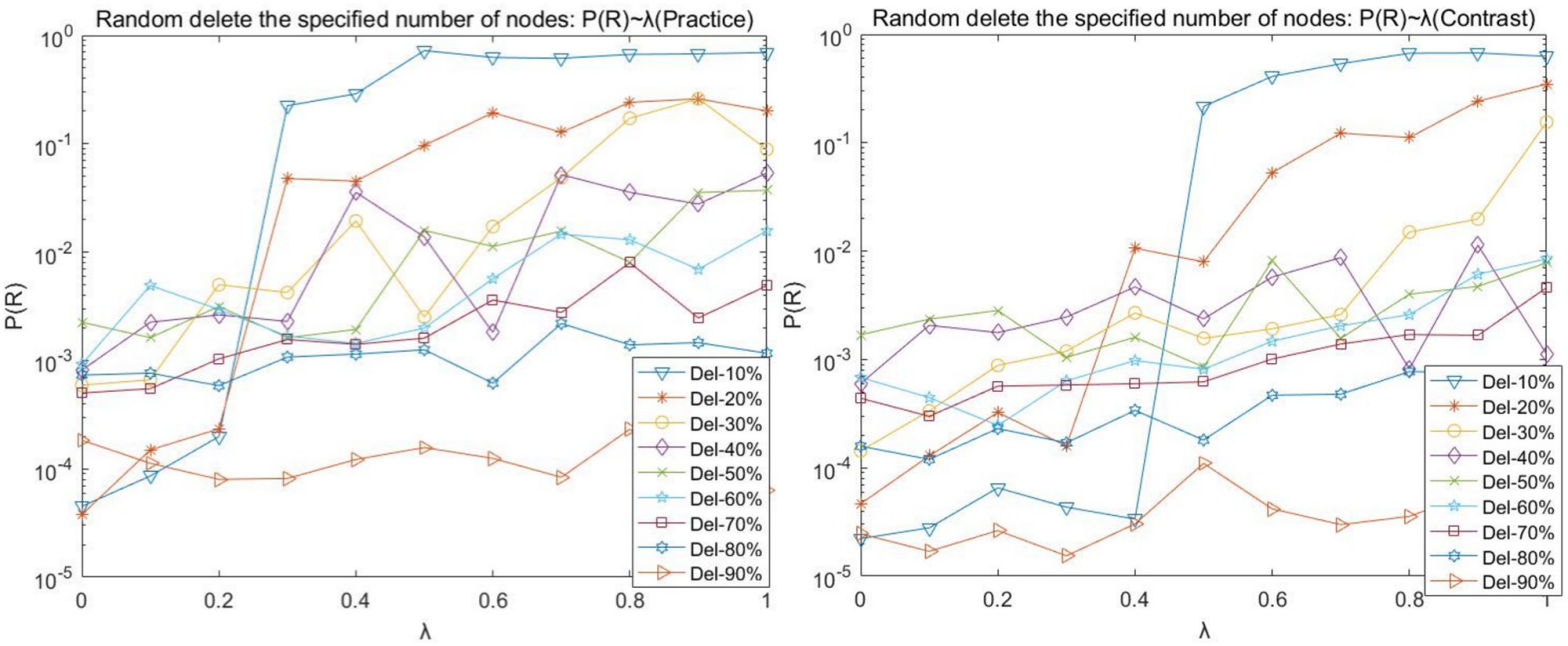
Random disruption- *P*(*R*) with λ variation diagram.

Figs 2–5 shows the curves of random disruption network P(N), P(L), P(R), and P(Q) with the variation of load factor λ. The left graph is the graph of the (Practice) network, and the right graph is the graph of the (Contrast) network.

We observed the plots of the (Practice) network in Figs 2–5 and found that

(A)There is an upper limit to the load of the network nodes. Under the same load factor λ (except λ = 0), the basic law of P(N), P(R), and P(Q) curves decreases as more nodes are deleted (with more randomness and occasional fluctuations); when the same number of nodes are deleted, the P(N), P(R), and P(Q) curves increase as the load factor λ increases.

(B)When the load factor λ = 0, the network cannot easily maintain the balance when the number of nodes is deleted instead. The P(N), P(R), and P(Q) values are the minimum values in the curve when 10% of nodes of the network are deleted, respectively. The impact of the network is relatively stable when the network reaches 30% of nodes deleted. (C)Comparing the P (N) curve in Fig 3 and the P (Q) curve in Fig 4 with the P (R) curve in Fig 5, we found that there are redundant and complicated lines in the network.

Our comparative observation of Figs 2–5 revealed that:

The trend of P(N), P(L), P(R), and P(Q) in the random disruption network varies consistently with the load factor λ = 0.

However, the network structure is dynamically robust to the impact of random disruption nodes on the network. Network (Contrast) requires a larger load factor to maintain better network robustness when the same number of nodes are randomly removed. Mainly, the proportion of intermediate nodes in the network (Contrast) increases, the strength of intermediate tier nodes becomes smaller relative to the network (Practice), and the maximum load capacity of intermediate tier nodes is smaller and prone to cascade failure phenomenon due to network node disruption.

(2)Target disruption analysis

**Fig 6:**
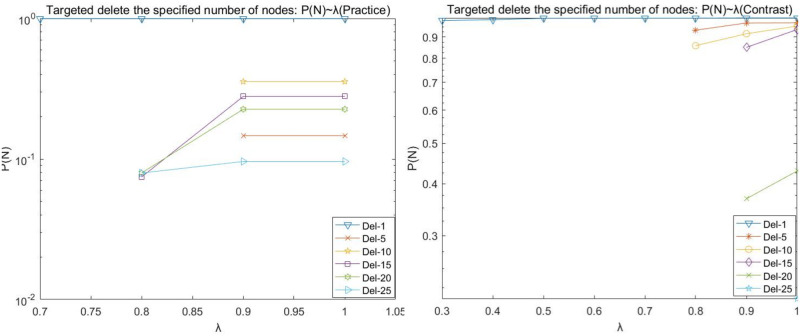
Target disruption- *P*(*N*) with λ variation diagram.

**Fig 7:**
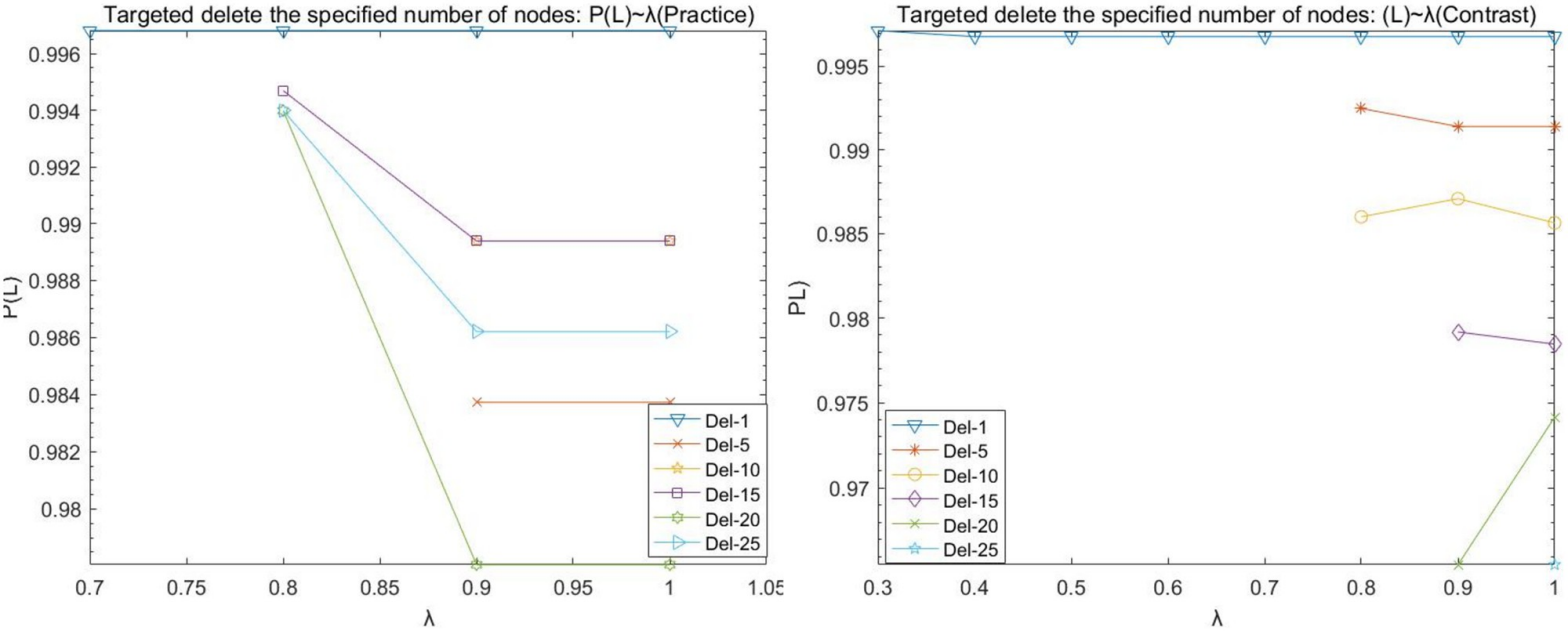
Target disruption- *P*(*L*) with λ variation diagram.

**Fig 8:**
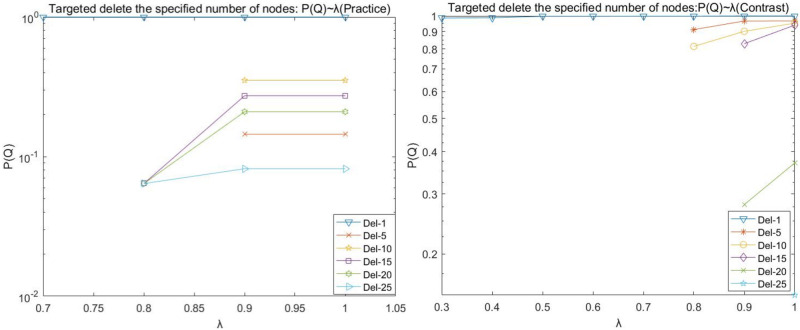
Target disruption- *P*(*Q*) with λ variation diagram.

**Fig 9:**
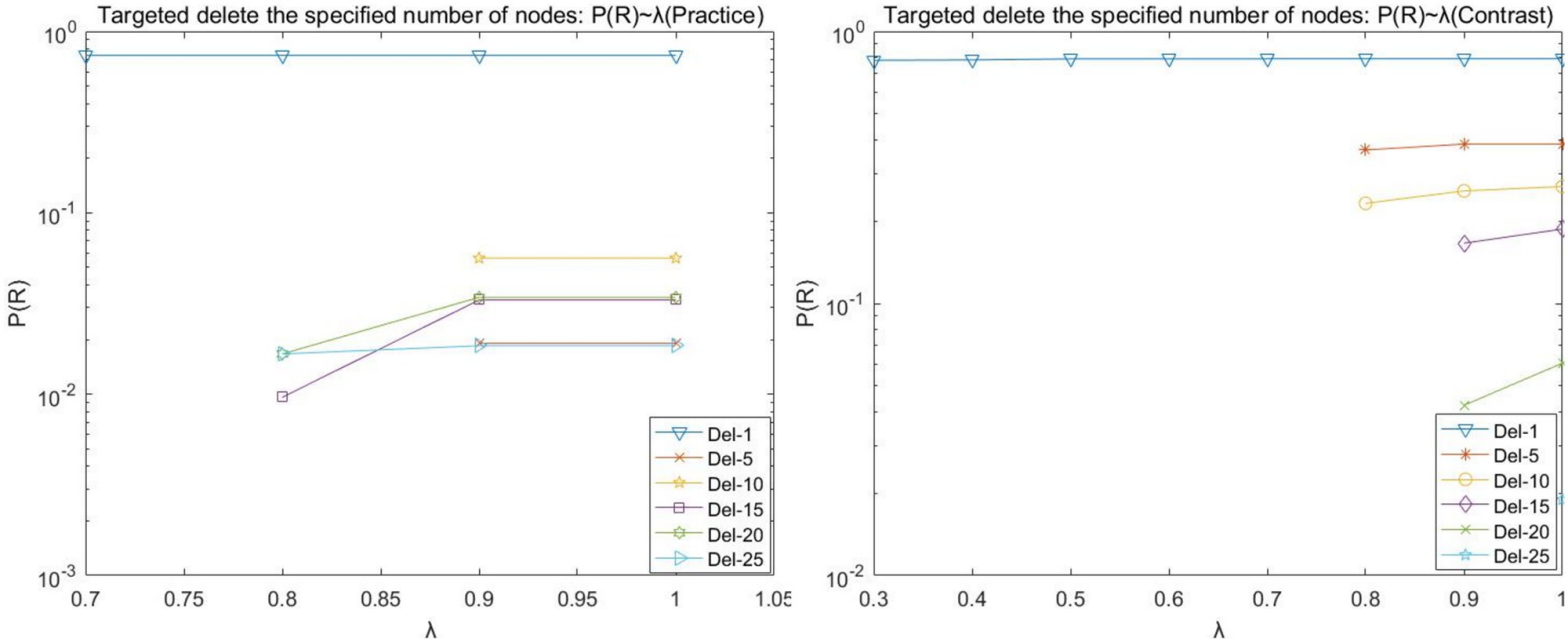
Target disruption- *P*(*R*) with λ variation diagram.

During the operation, it is found that the (Practice) network crashes at λ = 0 < 0.7 by deleting any number of nodes network, and the (Contrast) network crashes at λ = 0 < 0.3 by deleting any number of nodes network. Therefore, the load factor λ for network (Practice) in the line graphs depicted in Figs 6, 7, 8, and 9 is 0.4–1, and the load factor λ = 0 for network (Contrast) is 0.3–1.

We observed the plots of the (Practice) network in Figs 6–9 and found that target fresh cold chain network nodes, at λ < 0.7, attacking any number of nodes network is paralyzed by the occurrence of cascade failure. It is shown that attacking important nodes causes poorer network robustness when the network node load capacity is less than 1.7 times the node strength.

(A)The maximum number of nodes for which the network does not experience paralysis due to the removal of the target node at the load factor λ = 1 is 25 nodes for the (Practice) network and 25 nodes for the (Contrast) network.

(B)The (Practice) network has a higher strength due to fewer nodes in the intermediate level, and the network appears paralyzed due to the network cascade phenomenon when the dynamic robustness target deletes the nodes. Its line graph appears broken by folds, e.g., deleting the most important node, whose P(N), P(L), P(R), and P(Q) values are basically equal to the values of deleting 2, 3, 4, 5, and 10 target nodes when the load factor λ = 0.4; the whole network appears paralyzed when the load factor λ = 0.5−0.7, and the network has better robustness when the load factor λ reaches 0.8 and above. The main reason is that when the target node is deleted, as the node load factor increases, the upstream cascade failure of the nodes decreases, and the transaction volume transmitted downstream by the upstream nodes increases, while the maximum load of the downstream nodes is not enough to carry the transaction volume of the upstream nodes, and thus the phenomenon of node overload and failure occurs; when the load factor continues to increase until the maximum load of the downstream nodes is enough to carry the transaction volume of the upstream nodes, the network reaches better robustness.

(C)The (Contrast) network is not strong due to more nodes in the intermediate level. It requires a larger load factor to not experience paralysis due to the cascading phenomenon of the network once the number of target nodes is five or 10 when removed. The P(N), P(L), P(R), and P(Q) fold diagrams of the network (Contrast) do not show disruptions.

## Conclusion

The multi-layer complex network we have built with Chinese non-scale agricultural households as fresh agricultural product suppliers, and the impact of the disruption of China’s fresh cold chain network under the COVID-19 epidemic is analyzed as follows:

1. With the load factor λ increase, the robustness of the fresh cold chain network is enhancement. Load factor λ above 0.8, the network has strong robustness against random and target interrupts.
2. There are redundant and complicated lines in the multi-layer fresh cold chain network. Streamline the transportation route of agricultural products through network overall analysis: adjust the connection between network node enterprises, reallocate the weight of agricultural products transactions, and reduce the transportation route of goods.
3. The network disruption evolution of fresh cold chain is affected by the network structure. Random interrupt evolution: networks with relatively balanced node load capacity are greatly affected, such as the contract network in the article; Target interrupt evolution: the network will be greatly affected by the disruption of fewer nodes carrying large loads in the network, such as the practice network in the article.

## Supporting information

S1 Table Practice network data (https://osf.io/su5kv/). (XLSX)

S2 Table Contrast network data (https://osf.io/su5kv/). (XLSX)

## References

[1] Thadakamalla H P, Raghavan U N, Kumara S, et al. Survivability of multiagent—based supply networks: A topological perspective. EEE Intelligent Systems, vol. 19, no.5 pp.24–31, 2004. doi: 10.1109/MIS.2004.49

[2] Z. Gang, Y. Ying-Bao, B. Xu and P. Qi-Yuan. On the topological properties of urban complex supply chain network of agricultural products in mainland China. Transportation Letters The International Journal of Transportation Research, vol. 7, no.4, pp.188–195, 2015.

[3] Yang Li and Zi-ping Du. Agri-Food Suppy Chain Network Robustness Research Based on Complex Network. Proceedings of the 6thInternational Asia Conference on Industrial Engineering and Management Innovation (Chapter 92), pp.929-938, 2016.

[4] Barabasi A L, Albert R. Emergence of scaling in random networks. Science, vol. 286, no. 5439, pp.509–512, 1999. doi: 10.1126/science.286.5439.509 10521342

[5] Ghadge S, Killingback T, Sundaram B, Tran DA. A statistical construction of power-law networks. International Journal of Parallel, Emergent and Distributed Systems, vol. 25, no.3, pp.223–235, 2010. doi: 10.1080/17445760903429963

[6] Nguyen K, Tran DA. Fitness-based generative models for power-law networks. Optimization and Its Application, vol. 19, no.5 pp.39–53, 2012. doi: 10.1007/978-1-4614-0754-6_2

[7] Smolyarenko. Fitness-based network growth with dynamic feedback. Fitness-based network growth with dynamic feedback 10.1103/PhysRevE.89.042814 24827300

[8] Bell M, Perera S, Piraveenan M, Bliemer M, Latty T, Reid C. Network growth models: a behavioural basis for attachment proportional to fitness. Scientific Reporst, 7:42431, 2017. doi: 10.1038/srep42431 28205599 PMC5304319

[9] Supun Perera, Bell Michael G.H. and Bliemer Michiel CJ.. Network science approach to modelling the topology and robustness of supplychain networks: a review and perspective. Applied Network Science, 2017. 10.1007/s41109-017-0053-0 PMC6214257 30443587

[10] Nair A., Vidal J. M.. Supply Network Topology and Robustness Against Disruptions—An Investigation Using Multi-Agent Model. International Journal of Production Research, vol. 49, no.5 pp. 1391–1404, 2011. doi: 10.1080/00207543.2010.518744

[11] Hiroyasu Inoue, Yasuyuki Todo. Propagation of negative shocks across nationwide firm networks. PLOS ONE, vol. 14, no.3, 2019, 0213648. 10.1371/journal.pone.0213648 PMC6417701 30870470

[12] Yuhong Li, Christopher W. Zobel. Exploring supply chain network resilience in the presence of the ripple effect. Int. J. Production Economics, vol.228, 2020, 107693. doi: 10.1016/j.ijpe.2020.107693

[13] Shi Xiaoqiu, Long Wei, Li Yanyan, et al. Robustness of interdependent supply chain networks against both functional and structural cascading failures. Physica A, vol. 586, 2022, 126518. doi: 10.1016/j.physa.2021.126518

[14] Li G, Gu YG, Song ZH. Evolution of cooperation on heterogeneous supply networks. International Journal of Production Research, vol. 51, no.13, pp.3894–3902, 2013. doi: 10.1080/00207543.2012.754968

[15] Mari Sonia Irshad, Lee Young Hae, Memon Muhammad Saad, et al. Adaptivity of complex Network topologies for designing resilient supply Chain Networks. International Journal of Industrial Engineering, vol. 22, no.1, pp. 102–116 2015.

[16] Wang Y.; Zhang F. Modeling and analysis of under-load-based cascading failures in supply chain networks. Nonlinear Dyn, vol. 92, pp. 1403–1417 2018. doi: 10.1007/s11071-018-4135-z

[17] Zhang Y.; Yagan O. Robustness of Interdependent Cyber-Physical Systems against Cascading Failures. IEEE Trans. Autom. Control, vol. 65, pp. 711–726 2020. doi: 10.1109/TAC.2019.2918120

[18] Hosseinalipour S.; Mao J.; Eun D.Y.; Dai H. Prevention and Mitigation of Catastrophic Failures in Demand-Supply Interdependent Networks. IEEE Trans. Netw. Sci. Eng., vol. 7, pp. 1710–1723, 2019.

[19] Yang Q.; Scoglio C.M.; Gruenbacher D.M. Robustness of supply chain networks against underload cascading failures. Phys. A Stat. Mech. Appl., vol. 563, 2021, 125466. doi: 10.1016/j.physa.2020.125466

